# HIV Stigma and Discrimination in Colombian Healthcare: Insights from a National Cross-Sectional Analysis of General Practitioners

**DOI:** 10.3390/healthcare13091013

**Published:** 2025-04-28

**Authors:** Valentina Loaiza-Guevara, Juliana Paola Martinez Rivera, Juan Sebastian Castillo, Widad Dalel Gomez, Elisabet Deig Comerma, Juan S. Izquierdo-Condoy

**Affiliations:** 1 Facultad de Medicina, Fundación Universitaria Autónoma de las Américas, Pereira 660003, Colombia; 2 Facultad de Medicina, Universidad del Norte, Barranquilla 080003, Colombia; 3 Facultad de Medicina, Universidad del Quindío, Armenia 630001, Colombia; 4 Departamento de Medicina Interna, Hospital General de Granollers, 08402 Barcelona, Spain; 5 One Health Research Group, Universidad de las Americas, Quito 170125, Ecuador

**Keywords:** HIV infection, HIV-related stigma, healthcare professionals, general practitioners, Colombia

## Abstract

**Background/Objectives**: HIV-related stigma and fear among healthcare professionals represent significant barriers to effective patient care, contributing to delayed diagnosis and suboptimal treatment for people living with HIV (PLHIV). Although these issues have been studied in various settings, there is a lack of evidence on how they manifest among general practitionersin Colombia—a key group in the healthcare system. This study investigated the prevalence of stigma and fear among Colombian general practitioners and identified associated factors. **Methods**: A cross-sectional, observational study was conducted between February and May 2024, using a self-administered online survey targeting general practitioners. A validated 28-question instrument adapted from the Health Policy Project assessed fear, stigma, and related factors. **Results**: Among 579 participants, 68.6% exhibited low fear levels; however, specific clinical tasks, such as taking blood samples, elicited greater concern. Stigmatizing beliefs—such as associating HIV with irresponsible behavior—were reported by 25% of participants. Prior training in HIV care was inversely associated with stigma (OR = 0.681; 95% CI: 0.489–0.949), while older age and heterosexual orientation correlated with higher stigma levels. **Conclusions**: Despite generally low levels of fear and stigma, gaps in HIV-related training among physicians highlight the need for targeted educational initiatives. Promoting comprehensive HIV education in medical curricula and continuing professional development can reduce stigma, enhance confidence in care, and foster a more inclusive healthcare environment.

## 1. Introduction

Since the emergence of the human immunodeficiency virus (HIV) epidemic, widespread misconceptions regarding its transmission and the lived experiences of people living with HIV/AIDS (PLWHA) have fueled persistent stigma and discrimination [1,2]. HIV infection poses one of the greatest public health challenges globally due to its high transmissibility, continued growth, impact on morbidity and mortality, and the associated costs to the health system that further exacerbate the problem.

Healthcare professionals, particularly physicians, play a pivotal role in HIV prevention, diagnosis, and treatment. They are central to promoting early detection, educating patients on risk factors, and ensuring access to screening and care services [3]. Prejudices and stereotypes held by health professionals can influence the quality of care provided to PLWHA by discouraging them from seeking medical care. Stigma and discrimination among health workers have been documented in several countries across different regions of the world [4,5,6,7,8]. These attitudes result in underdiagnosis, poor adherence to treatment, and, ultimately, higher morbidity and mortality [9,10].

In addition to these clinical consequences, stigma from healthcare providers also affects the mental health of PLWHA, who experience higher rates of depression, social isolation, and suicidal ideation compared to the general population [11]. For instance, individuals in Serbia have reported that the most profound stigma they face originates from the healthcare sector itself [12]. Stigma within health services thus constitutes a critical barrier to both accessing and delivering effective HIV care [13].

Several studies have identified contributing factors to these stigmatizing attitudes among healthcare providers, including limited training in HIV care, unfounded fears of occupational exposure, moral or religious beliefs, and systemic gaps in education regarding HIV transmission and management [14,15,16,17]. These factors have led healthcare providers to spend less time with PLWH compared to other patients and to avoid physical contact during routine medical examinations [1,2,13,18,19,20]. The identification and recognition of stigma by healthcare workers are crucial strategies in the fight against HIV. Stigmatizing attitudes from key actors, such as healthcare providers, represent significant barriers to the well-being and quality of life of patients [4,5].

Despite the growing body of international literature on this topic, there is a notable lack of recent, empirical data from Colombia. National data indicate ongoing challenges in the HIV care cascade: in 2023, only 43.5% of newly diagnosed HIV cases in Colombia were identified in early stages, and only 68.35% of PLWHA reached an undetectable viral load, placing the country below global UNAIDS targets [21]. Given these gaps, a better understanding of physician-related stigma is essential to improve national HIV responses.

Despite the fundamental role of health workers in HIV management, this topic has not been widely explored in Colombia. This study aims to investigate the stigma and discrimination among general practitioners in Colombia towards HIV infection using a nationwide cross-sectional survey.

## 2. Materials and Methods

### 2.1. Study Design and Setting

This cross-sectional, observational study was conducted using a self-administered online survey targeting general practitioners in Colombia between February and May 2024. Colombia, a Latin American country, is bordered by the Pacific Ocean and shares borders with Ecuador, Peru, Brazil, Venezuela, and Panama, regions that have significant cultural influences [22]. According to the Colombian Ministry of Health, approximately 126,279 physicians were practicing in 2022, of whom 94,892 were general practitioners (75.1%) [23].

### 2.2. Population and Sample

The study population consisted of general practitioners residing in Colombia, defined as individuals holding a university degree in medicine who had not pursued or completed postgraduate specialization studies.

Based on a 2022 estimate of 94,892 general practitioners [23], the sample size was calculated using a 95% confidence level, a 5% margin of error, and a 50% response probability. The minimum required sample was 383 participants, determined using a standard formula for survey populations [24]:

$$n=\frac{\left(N.Z^{2}).(p.q\right)}{d^{2}.\left(N-1\right)+Z^{2}.p.q}$$

A non-probabilistic convenience sampling method was employed using the “Google Forms” platform. Participation was voluntary, and only responses from those who provided informed consent were included.

#### The Selection of Participants Was Based on the Following Criteria

Inclusion criteria: (1) being a general practitioner as defined above, (2) residing in Colombia at the time of the survey, and (3) accepting the informed consent before beginning the questionnaire. Exclusion criteria: (1) having completed or currently pursuing postgraduate medical specialization, (2) not residing in Colombia, or (3) declining to provide informed consent.

### 2.3. Questionnaire Development and Measurement

The study used a 28-question, self-administered online questionnaire adapted from the “Measuring HIV Stigma and Discrimination among People Working in Health Facilities” tool developed by the Health Policy Project [25]. The questionnaire was designed to evaluate fear and stigma related to HIV among general practitioners in Colombia.

The validation of the tool occurred in two stages. First, a public health expert reviewed the questionnaire for accuracy. Subsequently, a pilot test was conducted with 30 general practitioners, who provided feedback on comprehension and wording. Based on this, the final questionnaire in Spanish was structured into seven sections:

- Informed Consent;
- Sociodemographic Data (6 questions);
- Specialized Training on HIV (4 questions);
- Fear of HIV Infection (4 questions);
- Stigma and Discrimination Towards People Living with HIV (PLHIV) (6 questions);
- Stereotypes and Prejudices (7 questions).

### 2.4. Variables

The questionnaire collected a range of variables, including demographic data (e.g., sex, age, sexual orientation, workplace, and work experience). Additionally, it captured information on specialized training related to PLHIV, which encompassed training on HIV-specific care, infection control, care for key populations, and stigma and discrimination.

Using the framework of the Health Policy Project questionnaire and interpretation indicators proposed by UNAIDS, the instrument was designed as a 28-item, self-administered survey comprising closed-ended questions. Most items used four-point Likert-type scales (e.g., “not worried” to “very worried” or “strongly disagree” to “strongly agree”) to assess levels of fear, stigma, and attitudes toward PLHIV.

Fear of infection was assessed through four items evaluating participants’ emotional responses during specific work-related activities involving PLHIV. These were classified as noninvasive (e.g., touching clothes, taking temperature) or invasive (e.g., drawing blood, dressing wounds), based on infection risk. Participants who indicated worry on at least one invasive item were categorized as having a high level of fear [26].

Stigma and discrimination were assessed using items designed to detect stigmatizing beliefs and behaviors. Participants were classified as showing high stigma if they agreed with at least one stigmatizing statement. Additional items addressed manifestations of stigma, such as the use of unnecessary precautions (e.g., always wearing gloves for history-taking) and stereotypes about the behavior or morality of PLHIV.

Physicians’ attitudes toward key populations—including people who inject drugs, men who have sex with men, and sex workers—were also explored through items measuring willingness to provide care and personal comfort levels.

### 2.5. Data Collection and Management

Data collection was conducted using the online platform Google Forms, with participants accessing the survey through a unique link disseminated via social media channels, including Instagram and WhatsApp. The survey preamble provided an overview of the study objectives, reinforced the importance of confidentiality, and included a request for informed consent. Participants were required to agree to an electronic Participation Agreement before proceeding, ensuring anonymity throughout the process.

To ensure data integrity, all responses were subjected to a rigorous quality control process. This included verifying eligibility and consistency of responses, and identifying entries with implausible age ranges, indiscriminate selection of response options, duplicate submissions, or those that included only demographic data. Out of the 657 responses initially received, a total of 78 were excluded: 28 did not meet the selection criteria, 16 presented implausible age ranges, 17 showed indiscriminate selection of all options, 6 were identified as duplicates, and 11 included only demographic data. Ultimately, 579 valid responses were retained for inclusion in the final analysis.

### 2.6. Ethical Statement

This study adhered to the ethical principles established by the Declaration of Helsinki and complied with the protocols approved by the Ethics Committee of the Institute of Medical Diagnostics (IDIME) S.A. No personal, identifiable, high-risk, or sensitive information was collected during the study. All participants provided informed consent, ensuring full compliance with ethical research standards.

### 2.7. Statistical Analysis

Descriptive statistics were applied to analyze categorical variables, with frequencies and percentages calculated for each response. To explore the association between demographic, professional, and training characteristics and fear of HIV infection or stigma toward PLHIV, a logistic regression model was employed. Results were expressed as odds ratios (ORs) with 95% confidence intervals (CIs). Statistical significance was determined using a two-tailed approach, with a threshold of *p* < 0.05. All analyses were conducted using IBM SPSS Statistics for Windows, version 26.0 (IBM, Chicago, IL, USA).

## 3. Results

### 3.1. Sociodemographic and Professional Characteristics

A total of 579 general practitioners were included in the study. The majority were women (64.1%, *n* = 371), and 53.2% were aged between 27 and 59 years. Most participants identified as heterosexual (87.0%, *n* = 504). Regarding professional experience, 50.1% (*n* = 290) worked in outpatient services, and 45.4% (*n* = 262) reported having between one and five years of work experience. Only 11.9% (*n* = 69) had prior experience working in services specialized for people living with HIV (PLHIV), while 27.6% (*n* = 160) reported knowing someone close to them with an HIV diagnosis (Table 1).

### 3.2. Previous Training for PLHIV

**Figure 1:**
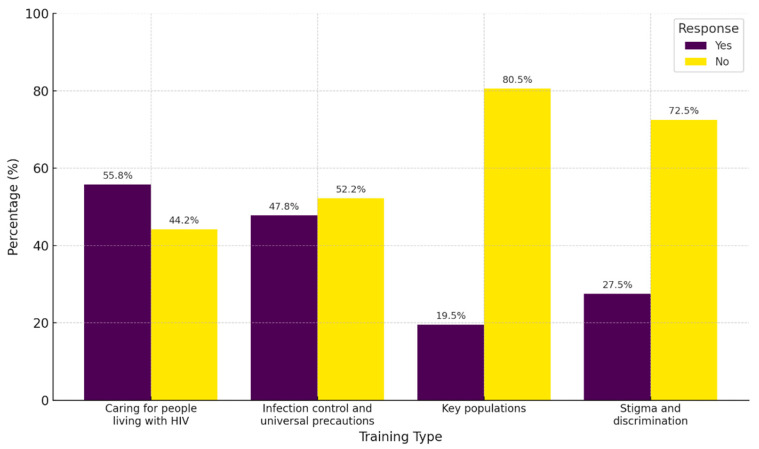
Health care training for PLHIV and key populations.

The analysis of previous training related to the care of PLHIV revealed that the highest proportion of training was associated with caring for PLHIV (55.8%), followed by training in infection control and universal precautions (47.8%). However, significant gaps in training were noted, particularly concerning key populations, where 80.5% of participants reported a lack of training. Similarly, 72.5% reported no training on stigma or discrimination toward PLHIV (Figure 1).

### 3.3. Fear of HIV Infection

**Figure 2:**
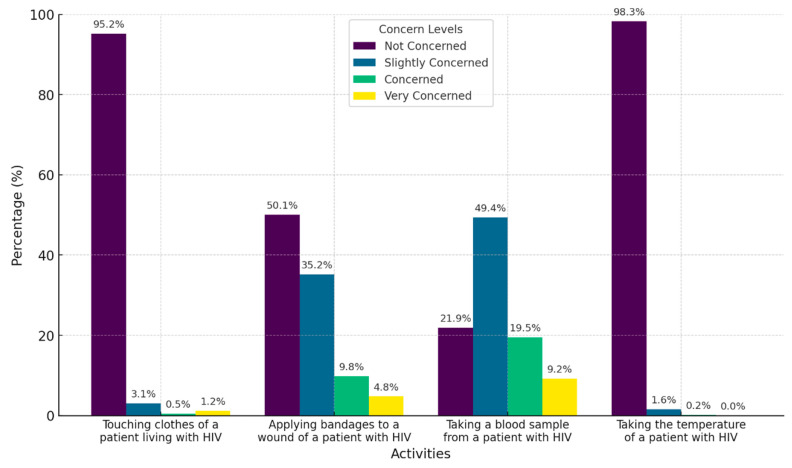
Characterization of Colombian physicians’ fear of HIV infection.

Fear of infection was assessed using four questions, with the highest level of fear identified in tasks such as taking blood samples from patients living with HIV (9.2% reported being very worried, 19.5% reported being worried). This was followed by applying bandages to a wound of a PLHIV (4.8% very worried, 9.8% worried). Activities eliciting the least fear included taking the temperature of a PLHIV (98.3% reported no concern) and touching their clothing (95.2% reported no concern) (Figure 2). Overall, the global evaluation showed that most participants exhibited minimal fear (65.6%, *n* = 397).

### 3.4. Factors Associated with Fear of HIV Infection

Among the sociodemographic factors studied, advanced age (27–59 years) was negatively associated with a high level of fear of infection (OR = 0.701; 95% CI: 0.493–0.998). Additionally, knowing someone close to them with an HIV diagnosis was also negatively associated with high fear (OR = 0.622; 95% CI: 0.411–0.941). Work characteristics further revealed that increased professional experience reduced the likelihood of high fear levels. Physicians with 5–10 years of work experience (OR = 0.517; 95% CI: 0.295–0.906) and those with over 10 years of experience (OR = 0.505; 95% CI: 0.274–0.931) exhibited significantly lower levels of fear (Table 2).

### 3.5. Stereotypes and Prejudices Toward PLWH

**Figure 3:**
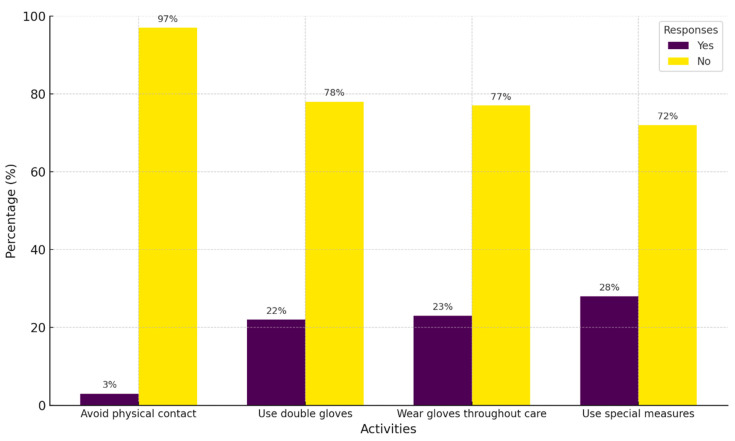
Characterization of the self-reported use of unnecessary precautions for HIV infection.

The evaluation of stereotypes and prejudices among physicians revealed that unnecessary precautions were relatively common. The most frequently reported behaviors included implementing special measures during care for PLHIV that were not applied to other patients (28.0%) and consistently using gloves throughout the care process, including during activities like taking a patient’s history or performing a physical examination (23%). In contrast, avoiding physical contact with PLHIV was the least reported behavior, with 97.0% of participants denying this practice (Figure 3).

**Figure 4:**
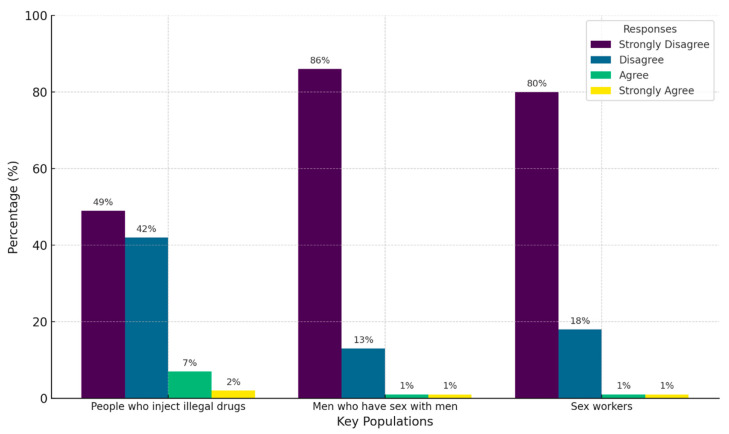
Characterization of physicians’ willingness and comfort in providing care to key populations.

Regarding physicians’ willingness and comfort in providing care to key populations, very little rejection was observed. Fewer than 2% of participants expressed strong agreement with preferring not to provide services to key populations. However, slightly higher reluctance was observed in relation to people who inject illicit drugs, with 7% of participants agreeing that they would prefer not to provide care to this group (Figure 4).

### 3.6. Stigma Among Physicians Toward PLWH

**Figure 5:**
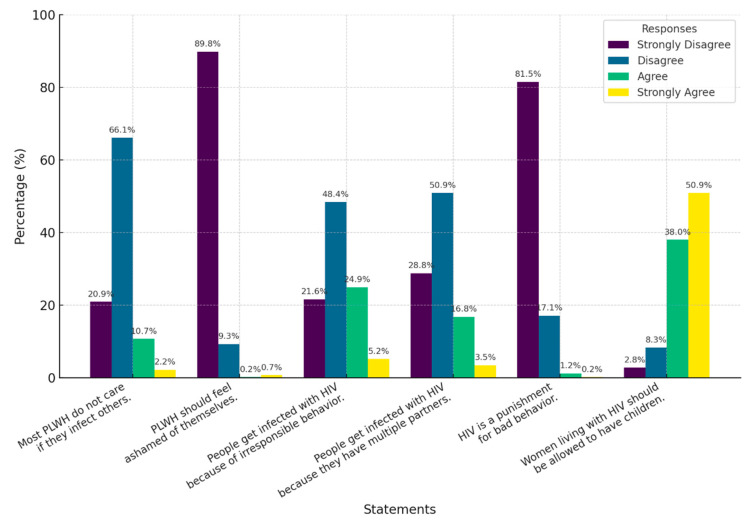
Characterization of the stigma of physicians towards PLWH.

The evaluation of stigma and prejudice revealed that most participants disagreed with stigmatizing statements. Specifically, 90% of respondents disagreed with the notion that PLWH should feel ashamed of themselves, and 82% rejected the idea that HIV is a punishment for bad behavior. However, 25% of participants agreed with the statement that people who contract HIV do so because they engage in irresponsible behavior. Additionally, 51% of respondents fully agreed that women living with HIV should be allowed to have children if they wish (Figure 5). In addition, the global assessment of stigma levels indicated that the majority of participating physicians (56.8%, *n* = 329) exhibited a low level of stigma toward PLWH, while 43.2% (*n* = 250) were classified as having a high level of stigma.

### 3.7. Factors Associated with Stigma Toward PLWH

Analysis of demographic variables showed that physicians aged between 27 and 59 years were significantly more likely to exhibit high levels of stigma toward PLWH (OR = 1.66; 95% CI: 1.206–2.356). Similarly, heterosexual participants were more likely to report high stigma levels (OR = 1.853; 95% CI: 1.100–3.120). In terms of professional characteristics, greater work experience was associated with higher stigma levels, particularly among physicians with 10 or more years of professional practice (OR = 2.145; 95% CI: 1.220–3.770). In contrast, previous training in caring for PLWH was the only factor associated with lower stigma levels, showing a negative association (OR = 0.681; 95% CI: 0.489–0.949) (Table 3).

## 4. Discussion

This study explored fear and stigma related to HIV infection among Colombian physicians, particularly in the context of their interactions with PLHIV. Several studies have employed the same standardized tool developed by the Health Policy Project to assess stigma among healthcare personnel in diverse contexts—including China, Thailand, and multi-country field trials across Africa, Asia, and the Caribbean—supporting the instrument’s cross-cultural applicability and reinforcing the comparability of our findings [27,28].

The sample was predominantly composed of female physicians, mirroring national trends of increasing female representation in medicine and medical training programs in Colombia and the broader Latin American region [29,30,31,32]. Most participants were young physicians aged 20 to 26 years, with limited professional experience of one to five years, likely influenced by the study’s reliance on self-reported electronic questionnaires, which are more accessible to younger populations. Additionally, 87% of participants identified as heterosexual, a pattern consistent with other studies among healthcare professionals [33,34].

A striking finding was the lack of specific training among more than 70% of physicians regarding the care of PLHIV and key populations. This is consistent with previous studies, such as Aziz M. et al., where 62% of surveyed physicians reported insufficient training on stigma-related issues, and Koseoglu Ornek et al., who found that 86% lacked specific education on HIV [2,20]. These results underscore an urgent need for academic and public health institutions in Colombia to prioritize HIV-focused training in medical curricula.

Regarding fear of HIV infection, 68.6% of participants reported low levels of fear, a smaller proportion compared to studies conducted in countries such as Indonesia and Yemen [35,36]. However, certain activities, such as applying bandages or taking blood samples from PLHIV, elicited higher levels of concern. These findings highlight the need to reinforce training on universal precaution practices, not only to protect healthcare workers but also to mitigate irrational fears. Moreover, the negative association between knowing someone diagnosed with HIV and fear supports the role of personal interactions in demystifying the disease and fostering positive attitudes, as observed in studies conducted in sub-Saharan Africa [19]. In addition, older age (27 to 59 years) and greater professional experience (five years or more) were also associated with reduced fear of HIV infection. This suggests that accumulated experience contributes to greater confidence in prevention measures and a more accurate understanding of HIV transmission routes. Similar findings in Egypt showed higher prejudice and fear among less experienced physicians [2]. The findings also revealed that some physicians adopted excessive protective measures, such as consistent glove use during non-invasive tasks. These practices reflect persistent misconceptions about HIV transmission, as standard universal precautions—such as hand hygiene, the use of gloves when in contact with blood or body fluids, and the safe handling of sharps—are sufficient for safely providing care to PLHIV [37]. Reinforcing these evidence-based hygiene practices may help reduce fear-driven and stigma-reinforcing behaviors within healthcare settings.

While the majority of physicians demonstrated low levels of stigma, approximately 20% agreed with stigmatizing beliefs, such as the idea that HIV is a consequence of irresponsible behavior or having multiple sexual partners. These beliefs oversimplify the multifaceted social, structural, and biological factors contributing to HIV transmission and risk perpetuating stigmas that can negatively impact patient care. Factors such as older age (27 to 59 years) and heterosexual orientation were associated with higher levels of stigma, consistent with studies showing that heterosexual individuals often display higher stigma rates [38,39]. These results may reflect cultural and generational norms, including the association of HIV with morally questionable behaviors, exacerbated by limited training on sexual diversity and insufficient knowledge of the structural drivers of HIV transmission. Addressing these cultural biases and promoting evidence-based education are critical to reducing stigma in these groups.

Training emerged as the only factor negatively associated with stigma toward PLHIV, reinforcing the pivotal role of education in reducing both fear and stigma, even among specialized groups like physicians. This finding aligns with systematic reviews by Okpua NC et al. and studies by Shahar E et al., which emphasize the effectiveness of educational programs in managing HIV-related stigma [6,40]. The high proportion of Colombian physicians untrained in caring for PLHIV and key populations [7] highlights modifiable factors that can be addressed through targeted interventions. Promoting ongoing training can create a stigma-free healthcare environment, with physicians playing a leading role in dismantling barriers to equitable care [8]. Beyond raising awareness, continuing education can challenge underlying attitudes that perpetuate stigma and discrimination, paving the way for a more inclusive and professional healthcare system [41,42].

### Limitations

This study provides valuable insights into fear and stigma toward HIV among Colombian general practitioners, yet several limitations should be considered when interpreting the findings. One notable limitation is the use of non-probability convenience sampling and online data collection, which may introduce selection and response biases, potentially excluding participants with limited access to electronic devices or the internet. To mitigate these challenges, a recruitment strategy was implemented, leveraging multiple dissemination channels, including medical associations, to reach a broader and more diverse sample.

Although the questionnaire was adapted from a validated standardized tool developed by the Health Policy Project, it was not revalidated psychometrically within the Colombian context. While a pilot test was conducted to ensure comprehension and cultural relevance, the absence of a formal validation process in this population constitutes a methodological limitation.

Additionally, the use of a closed-ended questionnaire limits the ability to capture nuanced attitudes or explanations behind certain responses, potentially constraining the depth of insight into physicians’ beliefs and behaviors.

Reliance on self-reported responses also poses a risk of recall bias or social desirability bias. To address this, the survey emphasized confidentiality and anonymity, employed neutral language, and highlighted the importance of honest responses to participants. These measures were designed to minimize biases and enhance the reliability and validity of the collected data.

## 5. Conclusions

This study found that most participants exhibited low levels of fear and stigma toward PLHIV. However, a substantial proportion of physicians lacked specialized training in caring for PLHIV and key populations, emphasizing the urgent need for targeted educational initiatives in medical curricula and continuing professional development.

Academic training emerged as the strongest factor associated with reduced stigma, underscoring its transformative role in shaping positive attitudes and fostering a more inclusive healthcare environment. These findings highlight the critical importance of integrating comprehensive HIV education into medical training programs to address stigma effectively.

Additionally, demographic and professional factors such as age, work experience, and personal familiarity with someone diagnosed with HIV were linked to lower levels of fear. This suggests that exposure and practical experience significantly contribute to reducing unfounded concerns and promoting confidence in managing HIV-related care. Conversely, stigmatizing beliefs were more prevalent among heterosexual and older physicians, reflecting underlying cultural and generational biases that require targeted intervention.

## Figures and Tables

**Table 1:** Sociodemographic and professional characteristics of the physicians participating in the study.

Characteristics		*n*	%
Sex	Male	208	35.9
	Female	371	64.1
Age (years)	20–26	267	46.1
	27–59	308	53.2
	60 or older	4	0.7
Sexual orientation	Heterosexual	504	87.0
	Non-heterosexual	75	13.0
Work setting	Academia/research	24	4.1
	Administrative	15	2.6
	Outpatient services	290	50.1
	Hospitalization	85	14.7
	Acute/critical care	165	28.5
Years of work experience	0–1	137	23.7
	1–5	262	45.3
	5–10	101	17
	More than 10	79	13.6
Experience in specialized HIV Units	No	510	88.1
	Yes	69	11.9
Knows someone with HIV diagnosis	No	419	72.4
	Yes	160	27.6

**Table 2:** Factors associated with fear of HIV infection among Colombian physicians.

		Fear of Infection				
		Low Fear (*n* = 397)		High Fear (*n* = 182)		OR (95% CI)
		*n*	%	*n*	%	
Demographic characteristics						
Age (years)	20–26 ref.	172	64.4%	95	35.6%	
	27–59	222	72.1%	86	27.9%	0.701 (0.493–0.998)
	60 or older	3	75.0%	1	25.0%	0.604 (0.062–5.883)
Sex	Female ref.	248	66.8%	123	33.2%	
	Male	149	71.6%	59	28.4%	1.253 (0.864–1.815)
Sexual orientation	Non-heterosexual ref.	57	76.0%	18	24.0%	
	Heterosexual	340	67.5%	164	32.5%	0.655 (0.373–1.148)
Knows someone with HIV diagnosis	No ref.	276	65.9%	143	34.1%	
	Yes	121	75.6%	39	24.4%	0.622 (0.411–0.941)
Professional characteristics						
Work setting	Academia/research ref.	17	70.8%	7	29.2%	
	Administrative	12	80.0%	3	20.0%	0.607 (0.130–2.836)
	Outpatient services	195	67.2%	95	32.8%	1.183 (0.747–2.950)
	Hospitalization	54	63.5%	31	36.5%	1.394 (0.521–3.733)
	Acute/critical care	119	72.1%	46	27.9%	0.939 (0.365–2.412)
Years of work experience (years)	0–1 ref.	82	59.9%	55	40.1%	
	1–5	181	69.1%	81	30.9%	0.667 (0.434–1026)
	5–10	75	74.3%	26	25.7%	0.517 (0.295–0.906)
	More than 10	59	74.7%	20	25.3%	0.505 (0.274–0.931)
Experience in specialized HIV units	No ref.	346	67.8%	164	32.3%	
	Yes	51	73.9%	18	26.1%	0.745 (0.422–1.315)
Training for the care of PLHIV						
Training on caring for people living with HIV	No ref.	229	70.9%	94	29.1%	
	Yes	168	65.6%	88	34.4%	1.276 (0.897–1.815)
Training on infection control and universal precautions	No ref.	201	66.6%	101	33.4%	
	Yes	196	70.8%	81	29.2%	0.822 (0.578–1.170)
Training on key populations	No ref.	318	68.2%	148	31.8%	
	Yes	79	69.9%	34	30.1%	0.925 (0.592–1.446)
Training on stigma and discrimination	No ref.	280	66.7%	140	33.3%	
	Yes	117	73.6%	42	26.4%	0.478–1.078)

**Table 3:** Factors associated with stigma towards PLWH among physicians.

		Stigma Level				
		Low Stigma (*n* = 329)		High Stigma (*n* = 250)		OR (95% CI)
		*n*	%	*n*	%	
Demographic characteristics						
Age (years)	20–26 ref.	170	63.7%	97	36.3%	
	27–59	157	50.8%	151	49.2%	1.66 (1.206–2.356)
	60 or older	2	50.0%	2	50.0%	1.753 (0.243–12.640)
Sex	Female ref.	218	58.8%	153	41.2%	
	Male	111	53.4%	95	46.6%	1.245 (0.885–1.753)
Sexual orientation	Non-heterosexual ref.	52	69.3%	23	30.7%	
	Heterosexual	277	55.0%	227	45.0%	1.853 (1.100–3.120)
Knows someone with HIV diagnosis	No ref.	236	56.3%	183	43.7%	
	Yes	93	58.1%	67	41.9%	0.929 (0.643–1.343)
Professional characteristics						
Work setting	Academia/research ref.	14	58.3%	10	41.7%	
	Administrative	8	53.3%	7	46.7%	1.225 (0.334–4.491)
	Outpatient Services	172	59.3%	118	40.7%	0.960 (0.413–2.235)
	Hospitalization	43	50.6%	42	49.4%	1.367 (0.547–3.418)
	Acute/critical care	92	55.8%	73	44.2%	1.111 (0.466–2.645)
Years of work experience (years)	0–1 ref.	88	64.2%	49	35.8%	
	1–5	152	58.0%	110	42.0%	1.300 (0.848–1.992)
	5–10	53	52.5%	48	47.5%	1.626 (0.963–2.747)
	More than 10	36	45.6%	43	43.2%	2.145 (1.220–3.770)
Experience in specialized HIV Units	No ref.	293	57.5%	217	42.5%	
	Yes	36	52.2%	37	47.8%	1.238 (0.748–2.049)
Training for the care of PLHIV						
Training on caring for people living with HIV	No ref.	132	51.6%	124	48.4%	
	Yes	197	61.0%	126	39.0%	0.681 (0.489–0.949)
Training on infection control and universal precautions	No ref.	163	54.0%	139	46.0%	
	Yes	166	59.9%	111	40.1%	0.784 (0.564–1.091)
Training on key populations	No ref.	256	54.9%	210	45.1%	
	Yes	73	64.4%	40	35.4%	0.668 (0.436–1.023)
Training on stigma and discrimination	No ref.	231	55.0%	189	45.0%	
	Yes	98	61.6%	61	38.4%	0.761 (0.524–1.105)

## Data Availability

The original contributions presented in this study are included in the article. Further inquiries can be directed to the corresponding author.

## Abbreviations

The following abbreviations are used in this manuscript:

HIV	Human immunodeficiency virus
PLWHA	People living with HIV/AIDS
PLHIV	People living with HIV
CI	Confidence interval
OR	Odds ratio
UNAIDS	Joint United Nations Programme on HIV/AIDS
